# Basolateral Amygdala Lesion Inhibits the Development of Pain Chronicity in Neuropathic Pain Rats

**DOI:** 10.1371/journal.pone.0070921

**Published:** 2013-08-05

**Authors:** Zheng Li, Jing Wang, Lin Chen, Meng Zhang, You Wan

**Affiliations:** 1 Neuroscience Research Institute, Peking University, Beijing, P. R. China; 2 National Key Laboratory of Cognitive Neuroscience and Learning, School of Brain and Cognitive Sciences, Beijing Normal University, Beijing, P. R. China; 3 Key Laboratory for Neuroscience, Ministry of Education/National Health and Family Planning Commission, Peking University, Beijing, P. R. China; University of Arizona, United States of America

## Abstract

**Background:**

Chronicity of pain is one of the most interesting questions in chronic pain study. Clinical and experimental data suggest that supraspinal areas responsible for negative emotions such as depression and anxiety contribute to the chronicity of pain. The amygdala is suspected to be a potential structure for the pain chronicity due to its critical role in processing negative emotions and pain information.

**Objective:**

This study aimed to investigate whether amygdala or its subregions, the basolateral amygdala (BLA) and the central medial amygdala (CeA), contributes to the pain chronicity in the spared nerve injury (SNI)-induced neuropathic pain model of rats.

**Methodology/Principal Findings:**

(1) Before the establishment of the SNI-induced neuropathic pain model of rats, lesion of the amygdaloid complex with stereotaxic injection of ibotenic acid (IBO) alleviated mechanical allodynia significantly at days 7 and 14, even no mechanical allodynia at day 28 after SNI; Lesion of the BLA, but not the CeA had similar effects; (2) however, 7 days after SNI when the neuropathic pain model was established, lesion of the amygdala complex or the BLA or the CeA, mechanical allodynia was not affected.

**Conclusion:**

These results suggest that BLA activities in the early stage after nerve injury might be crucial to the development of pain chronicity, and amygdala-related negative emotions and pain-related memories could promote pain chronicity.

## Introduction

Neuropathic pain is a kind of chronic pain resulting from injury of somatosensory system. It is characterized by pain persistence even after original damages have been recovered [1]. Its symptoms include allodynia, hyperalgesia and spontaneous pain. Many studies have demonstrated that several brain regions contribute to chronic pain by showing that pain is reduced after inactivation of brain regions at the late stage of chronic pain [2], [3].

One of the most obvious characteristics of neuropathic pain is its chronicity and persistence. Chronic pain is a process that develops from somatosensory nerve injury and then maintains. So, if we want to understand why pain symptoms last for a long time after nerve injury, it is not enough to put focus on only the stage when chronic pain has been established. Instead, it is necessary to obtain experimental data at multiple time points during the development and maintenance of chronic pain. After peripheral nerve injury, plastic changes occur only at early stage but not at late stage, indicating that supraspinal structures of the central nervous system might be responsible for sustained pain at late stage [4], [5]. In addition, clinical data demonstrate that negative emotions such as depression and anxiety are high risk factors in the development of chronic pain, and promote transition from acute pain to chronic pain [5]. So, it is reasonable to speculate that supraspinal brain regions which process negative emotional information probably contribute to pain chronicity or facilitate the development of chronic pain.

The amygdala, a brain region in the limbic system, is well known for its important roles in negative emotions including depression and anxieties [6], [7], [8]. It processes nociceptive information and undergoes neuroplasticity in neuropathic pain [9]–[12]. Therefore, it is considered as a center where the negative emotional information and the nociceptive information are integrated [13]. Activation of the amygdala either increases or inhibits acute pain and experimental pain. However, the role of amygdala in chronic pain is still unknown. Based on the theories of pain chronicity and functions of the amygdala, we hypothesize that the amygdala promotes the pain chronicity.

In the present study, we investigated the effect of the amygdala lesion in the early or the late period of spared nerve injury (SNI) on the development of pain chronicity. Considering that the sub-nuclei of the amygdala have different functions, we further investigated the role of the basolateral amygdala (BLA) and the central amygdala (CeA) in pain chronicity separately.

## Materials and Methods

### Ethics Statement

Adult, male Sprague-Dawley rats weighing 250 to 300 g were used. They were provided by the Department of Experimental Animal Sciences, Peking University Health Science Center. All rats were housed individually in cage at temperature 22±1°C and raised under a natural light/dark cycle. Food and water were available *ad libitum*. All experimental procedures were in accordance with the Guidelines of International Association for the Study of Pain and were approved by the Animal Care and Use Committee of Peking University Health Science Center. The behavioral experiments remained double blind.

### Establishment of Neuropathic Pain Model of Rats with Spared Nerve Injury (SNI)

SNI neuropathic pain model of rats was established as described in previous report [14]. Rats were anesthetized with 1% pentobarbital sodium (50 mg/kg, *i.p.*), then the left sciatic nerve and its trifurcations were exposed. The common peroneal nerve and the tibial nerve were tightly ligated with 5.0 silk thread, sectioned distal to the ligation with removal of 2–4 mm nerve stump. The sural nerve was kept intact. Muscle and skin were closed in two layers under sterile operation. Only animals that developed mechanical allodynia were used. For sham operated rats, the left sciatic nerve and its trifurcation were exposed but not manipulated.

### Behavioral Test for Mechanical Allodynia

Before the behavioral test, all rats were allowed to acclimate for 1 h each day in the test environment for 3 days. Rats were placed on a wire-mesh floor with perspex cage and habituated to the environment for 20 min before experiment. *Von* Frey filaments (Stoelting, U.S.A, 0.41–15.1 g) were applied to the lateral plantar surface of the hind paw in the receptive field of the sural nerve. The 50% paw withdrawal threshold (PWT) in response to a series of *von* Frey filaments was measured by the “up and down” method as described by Chaplan *et al.* [15]. For control, only rats with basal paw withdrawal threshold equal to 15.1 g were recruited in the further experiments.

### Amygdala Lesion with Ibotenic Acid (IBO)

The rats were anesthetized as mentioned above and fixed in a stereotaxic apparatus (Stoelting, U.S.A). For the operation of amygdala complex lesion, IBO (Sigma-Aldrich, U.S.A) was injected at four sites using following coordinates: CeA [anterial-posterial (AP), −2.0; medial-laterial (ML), ±4.1; dorsal-ventral (DV), −7.7 relative to bregma], BLA (AP, −2.9; ML, ±4.8; DV, −8.1 relative to bregma). IBO at 2 µg dissolved in 0.2 µl normal saline (NS) was infused at a rate of 0.1 µl/min, using a 0.5 µl-syringe (Gaoge, China) connected to an infusion pump (World Precision Instruments, U.S.A) at each site. The syringe was left in place for 1 min after infusion to allow the IBO diffusion. Amygdala sham lesion was made similarly to the IBO lesion with an infusion of 0.2 µl normal saline instead. For CeA lesion or BLA lesion, IBO was injected bilaterally at either CeA or BLA.

### Experiment Procedure

**Figure 1:**
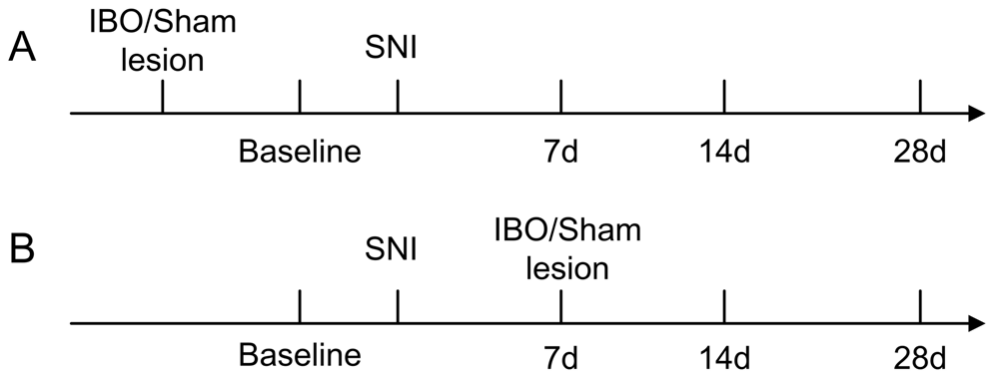
Procedure of SNI lesion and behavioral test for mechanical allodynia. (A) IBO lesion before SNI model establishment. The amygdaloid complex or the BLA or the CeA was lesioned first, then SNI surgery was performed. PWT in response to *von* Frey filament stimulation was measured before SNI as baseline and 7, 14, 28 days after SNI. (B) SNI model establishment before the IBO lesion. The amygdaloid complex, or the BLA or the CeA, was lesioned separately at day 7 after SNI. PWT was measured before SNI as baseline and 7, 14 and 28 days after SNI.

We used two different experimental procedures as shown in the schematic diagram in Fig. 1. For the first procedure, amygdala lesion was performed before the establishment of the SNI neuropathic pain model; for the second procedure, amygdala lesion was performed 7 days after the establishment of the SNI model.

### Histology Confirmation of Lesion Locations in Amygdala

**Figure 2:**
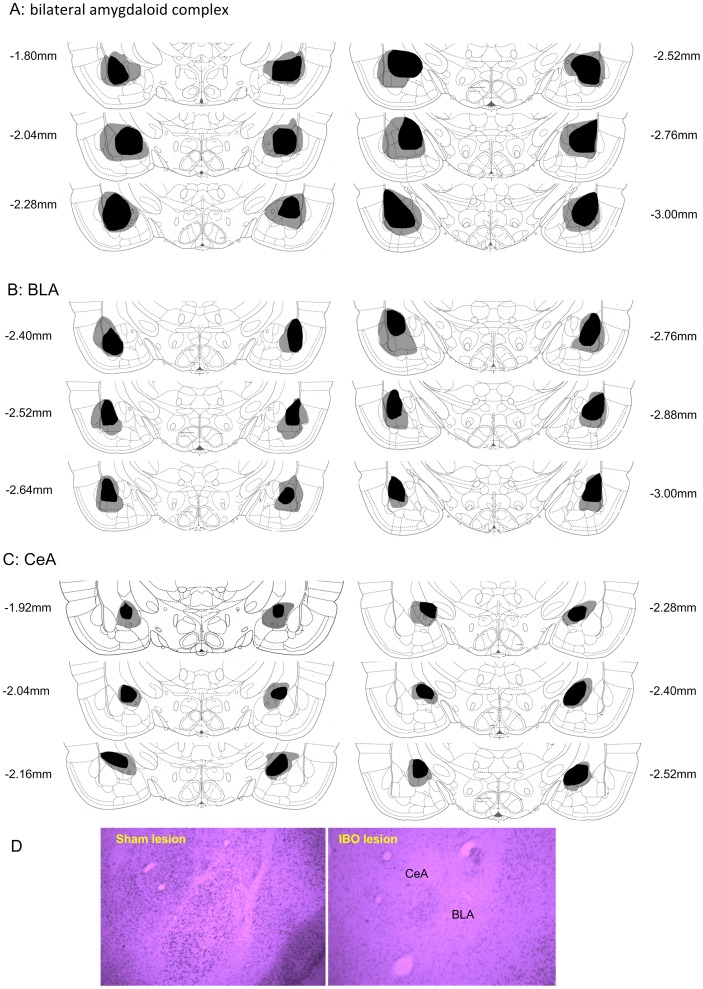
Schematic representation of the maximum (grey zone) and the minimum (black zone) extent of lesion to the bilateral amygdala. Re-construction are presented on a series of coronal sections according to the stereotaxic atlas of Paxinos and Watson [30]. (A) Amygdaloid complex; (B) BLA; (C) CeA; (D) Photograph of coronal sections of the amygdala in sham lesion (left) and IBO lesion (right) rats. Note that loss of cell population and cell shrinkage in the regions of CeA and BLA in the IBO-lesioned rats.

After behavioral test, rats were anesthetized with pentobarbital sodium (50 mg/kg, *i.p.*) and perfused via aorta with 300 ml normal saline and 300 ml 4% paraformaldehyde solution in 0.1 M phosphate buffer (PB, pH 7.4). Brain samples were stored in 4% paraformaldehyde overnight and then sequentially in 20% and 30% sucrose solution in PBS (pH 7.2) for 24 h. Coronal brain sections were cut at 30 µm and stained with Nissl to confirm the lesion locations in the amygdala by microscopic inspection. Fig. 2 shows the schematic illustration of damages in the amygdaloid complex, the CeA and the BLA in all rats.

### Statistical Analysis

Data are expressed as mean ± SEM. Two-way analysis of variance with time point as repeated measures (two-way mixed-model ANOVA) was used. When the interaction effect is significant, Bonferonni *post hoc* test was used to compare PWTs between groups. *p*<0.05 was considered as statistically significant.

## Results

### Amygdala Lesion before SNI Inhibited the Development of Mechanical Allodynia in SNI Rats

This part aims to investigate whether the amygdaloid complex is crucial to the initiation of neuropathic pain. Bilateral CeA and BLA were lesioned either by IBO or sham-lesioned by normal saline injection. Seven days after the lesion, SNI surgery or sham surgery was performed in each individual rat. Mechanical allodynia, which is represented by PWTs in response to mechanical stimulation, was measured before surgery and 7, 14, 28 days after surgery.

**Figure 3:**
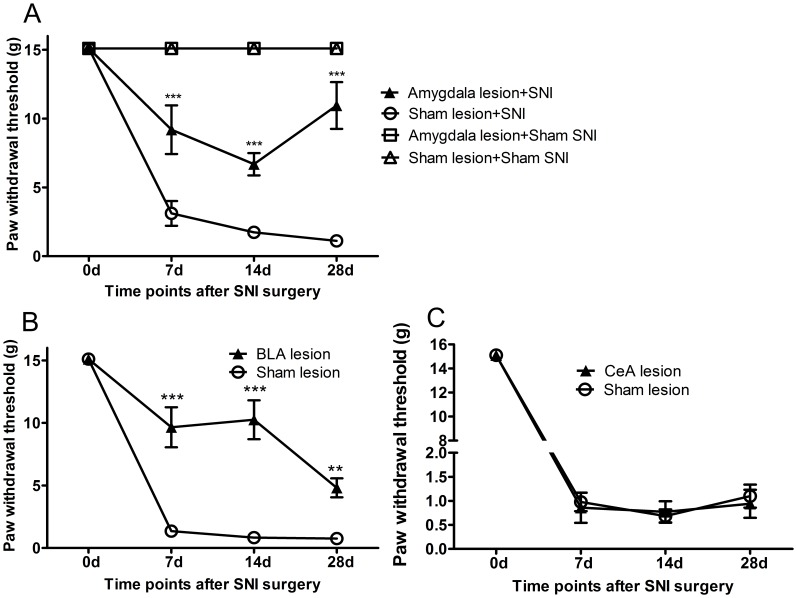
Effects of amygdala lesion before the SNI surgery on mechanical allodynia. (A) Amygdaloid complex lesion before SNI increased PWT at days 7, 14 and 28 after SNI, compared to amygdaloid complex sham lesion. (B) Basolateral amygdala (BLA) lesion before SNI increased PWTs at days 7, 14 and 28 after SNI, compared to BLA sham lesion. (C) Central amygdala (CeA) lesion before SNI did not change PWTs compared to the sham lesion. * p<0.05, ** p<0.01, *** p<0.001: SNI rats with IBO lesion compared with the SNI rats with sham lesion by Bonferonni *post hoc* test. n = 8–9.

As shown in Fig. 3A, PWT to mechanical stimulation was significantly different among groups over all time points (interaction effect, F_ (9,84)_ = 25.11, *p*<0.001; time as main factor, F_(3,84)_ = 55.65, *p*<0.001; group as main factor, F_(3,84)_ = 136.83, *p*<0.001). In the sham SNI rats with IBO lesion or with sham lesion, PWTs had no significant difference at all time points (*p*>0.05), indicating that amygdala lesion had no effect on basal pain threshold.In the SNI rats, before the SNI surgery, there is no significant difference between PWTs in the SNI rats with IBO lesion or with sham lesion (*p*>0.05). However, after the SNI surgery, SNI rats with IBO-lesion or with sham-lesion of amygdala showed different responses to mechanical stimulation, *i.e.*, SNI rats with amygdala IBO-lesion showed much higher PWTs compared to SNI rats with amygdala sham-lesion at days 7, 14 and 28. These results suggest that amygdala IBO-lesion could alleviate mechanical allodynia in SNI rats when amygdala was lesioned before the SNI surgery.

To further distinguish the role of two sub-regions of the amygdaloid complex, the BLA or the CeA was separately lesioned with the same procedure as in the above. Effect of the BLA IBO-lesion was different to the CeA IBO-lesion on neuropathic pain in SNI rats. The effect of the BLA IBO-lesion was very similar to the amygdaloid complex IBO-lesion. BLA IBO-lesion rats had much higher PWTs than sham-lesion rats over time (interaction, F_(3, 48)_ = 13.29, *p*<0.001; time as main factor, F_(3,24)_ = 83.74, *p*<0.001; group as main factor, F_(1,48)_ = 79.98, *p*<0.001) (Fig. 3B). BLA lesion significantly reduced mechanical allodynia in the SNI rats at days 7, 14 and 28 after SNI surgery. However, the CeA IBO-lesion did not have any significant effect on the PWTs compared to the sham-lesion (group as main factor, F_(1, 48)_ = 0.09, *p*>0.05) (Fig. 3C).

### Amygdala Lesion after SNI had no Effect on the Development of Mechanical Allodynia in SNI Rats

**Figure 4:**
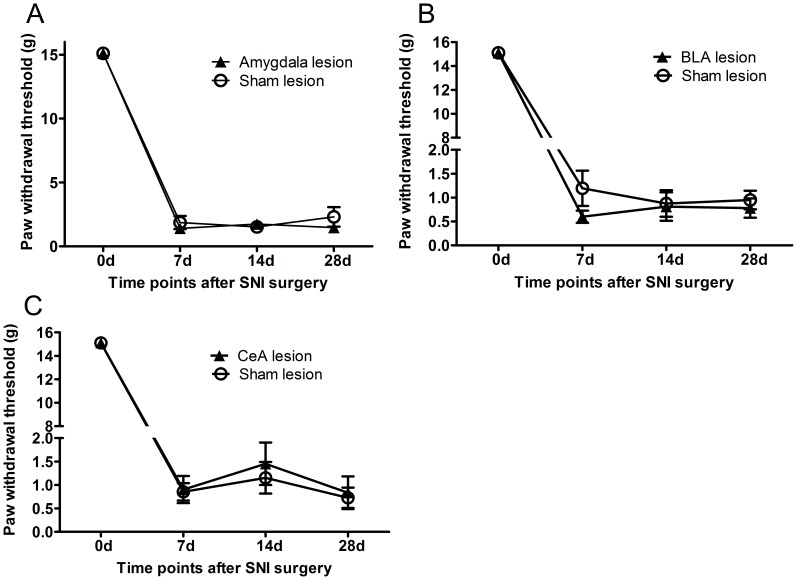
Effects of amygdala lesion 7 days after the SNI surgery on mechanical allodynia. Amygdaloid complex lesion (A), basolateral amygdala (BLA) lesion (B), or Central amygdala (CeA) lesion (C) had no obvious effect on PWT compared with the sham lesion. n = 7–9.

To investigate the effect of the amygdala in the maintenance of neuropathic pain, we established the SNI model of rat first, then lesioned the amygdala. At day 7 after the SNI surgery, the amygdaloid complex was lesioned with IBO stereotaxic injection. PWTs were measured at each time point. Results are shown in Fig. 4. We can see that the IBO-lesion of the amygdaloid complex, the CeA or the BLA had no significant effect on mechanical allodynia in SNI rats. PWTs still decreased at day 7 to day 28 in SNI rats in three IBO-lesioned groups of the amygdaloid complex (Fig. 4A), the CeA (Fig. 4B) or the BLA (Fig. 4C) as in the sham-lesioned group (amygdaloid complex, group as main factor F_(1,42)_ = ,0.62 *p*>0.05; BLA, group as main factor F_(1,42)_ = 0.93, *p*>0.05; CeA, group as main factor F_(1,42)_ = 0.18, *p*>0.05). These results suggest that neither the amygdaloid complex nor its sub-regions (the BLA or the CeA) influences mechanical allodynia once neuropathic pain has been established.

## Discussion

### The Amygdala Promotes the Development of Chronic Pain in the Early Stage after Nerve Injury

It is well known that the amygdala is involved in the pain-related emotional processing of anxiety, fear and fear memory. These negative emotions are high risk factors for pain persistence. Thus, we speculate that the amygdala is very likely to play a role in pain chronicity. In the present study, we found that 7 days after nerve injury, *i.e.* when chronic pain had been established, the amygdala lesion (either CeA or BLA) did not influence the development of chronic pain. While before the nerve injury, *i.e.* before chronic pain was established, amygdala lesion significantly inhibited the persistence of pain. These results indicate that the amygdala indeed participates in chronic pain, but only at certain period. At the early stage, amygdala is crucial for the formation of chronic pain. This time-dependent characteristic of the amygdala in chronic pain is supported by the fact that early stage is critical time window for chronic pain management [16].

Amygdala hyperactivity was observed at very early stage of neuropathic pain in SNL rats in an electrophysiological experiment [11]. Recent studies reported that functional interactions between the amygdala and the anterior cingulate cortex or the prefrontal cortex are involved in pain-related negative emotions and cognition [17], [18]. Amygdala blockade by the IBO lesion at the very early stage probably inhibited the initiation of these amygdala-related inter-brain region interactions, then reduced negative emotions, and thus could interfere with pain perception and pain chronicity. In addition, enhanced synaptic transmission between parabrachial nuclei–CeA and CeA–BLA was observed in rats with neuropathic pain [19]. It is well known that the neural plasticity in the BLA is very important in fear memory [20]. The amygdala also processes pain sensory information [21]. Therefore, one possibility is that the amygdala processes pain information and forms pain-related memory template at the early stage after nerve injury. On the contrary, at the late stage in chronic pain after nerve injury, *i.e.* when pain memory has been established and when the vicious circle has begun between negative emotions and pain, the amygdala lesion hardly prevents chronic pain development and maintenance. Additionally, as previous finding suggested that hypertrophy and cell proliferation in the CeA and the BLA is associated with depression-like behavior in SNI neuropathic pain rats [12], blockage of amygdala activity may alleviate depression, which in return to facilitate the development of chronic pain [5]. Compensation is another possible mechanism to explain these brain region-lesion results. Several previous studies reported that the excitotoxic lesion of one brain area could induce changes of neuropeptides [22], ganglioside [23] and neurotransmitters [24] in areas which have connections with the lesioned area several days after the lesion. So, we speculate that brain areas which have connections with amygdala, such as the ACC and the PFC, may undergo compensatory changes after the amygdala lesion. This is also an underlying mechanism for reduced allodynia in our results.

Considering that amygdala lesion could increase, but not decrease the locomotor activities [25], it is hard to image that the amygdala lesion contributes to the decreased pain withdrawal response in SNI rats.

### The BLA of the Amygdala is Responsible for the Development of Chronic Pain

SNI rats with the BLA IBO-lesion did not exhibit the expected persistent pain. By contrast, SNI rats with the CeA lesion still developed chronic pain. The BLA and the CeA have distinct functions in pain processing, learning and memory. The BLA is essential in the fear learning system [19], [26]. It contributes to the storage of the fear memory. The CeA was involved in the acquisition and the expression of fear conditioning [7], [8], [27]. Due to BLA activation after nerve injury, the pain-related memory template was established. Even without further nociceptive inputs when the nerve injury had been cured, this pain memory template could still produce pain perception. In this context, our results may suggest that the pain-related memory is crucial to the chronic pain development [28], [29]. In addition, the CeA is considered as “the nociceptive amygdala”, which processes nociception [15], [25]. The BLA is considered as a center for emotional processing [7], [8], indicating that the chronicity of pain is not related to nociception itself but related to emotion. Accordingly, our results provide an evidence that the affective and cognitive aspects of pain outweigh the sensory one in the development of chronic pain [29]. Although the CeA also underwent neural plasticity and modulated hyperalgesia in chronic pain [19], it did not show influence on pain chronicity in our experiment. Actually, the inconsistency between our results and others suggest that hyperalgesia and sustained pain may have distinct mechanisms, which needs further investigation. Another possible reason is that the BLA deals with body pain while the CeA modulates visceral pain [28], [29]. At day 28 after SNI, the pain intensity in the BLA IBO-lesioned rats was not as low as in the amygdala IBO-lesioned rats, suggesting that fibers connecting the CeA with the BLA are functionally important.

Our study is interesting but preliminary. We extended the function of the BLA to the pain chronicity, but the underlying mechanisms need further investigation. Although mechanical allodynia is the most stable feature in SNI neuropathic pain rats, the effect of BLA lesion on other submodalities in neuropathic pain rats, or the effect of the lesion on other models of neuropathic pain still deserve further investigation. Also it will be valuable to study the processing in the interaction between pain information and negative emotions in the BLA.

In conclusion, we report a novel mechanism for the development and chronicity of neuropathic pain. The BLA, but not the CeA of the amygdala, lesion prior to the nerve injury could inhibit the chronicity of neuropathic pain. These results indicate that BLA activities at the early stage after the nerve injury may play an important role in the development of chronic pain. Our results also give a prompt that amygdala-related negative emotions and pain memories may contribute to the pain chronicity, and the amygdala is a potential region of interest for preventing the development of chronic pain in the early stage after the nerve injury.
